# Mixed Ionic-Electronic Conductivity, Redox Behavior and Thermochemical Expansion of Mn-Substituted 5YSZ as an Interlayer Material for Reversible Solid Oxide Cells

**DOI:** 10.3390/ma14030641

**Published:** 2021-01-30

**Authors:** Alejandro Natoli, Blanca I. Arias-Serrano, Enrique Rodríguez-Castellón, Agnieszka Żurawska, Jorge R. Frade, Aleksey. A. Yaremchenko

**Affiliations:** 1 CICECO–Aveiro Institute of Materials, Department of Materials and Ceramic Engineering, University of Aveiro, 3810-193 Aveiro, Portugal; anatoli@ua.pt (A.N.); blanca@ua.pt (B.I.A.-S.); jfrade@ua.pt (J.R.F.); 2 Leibniz Institute for Plasma Science and Technology, Felix-Hausdorff-Street 2, 17489 Greifswald, Germany; 3 Department of Inorganic Chemistry, Faculty of Science, University of Malaga, Campus de Teatinos, 29071 Málaga, Spain; castellon@uma.es; 4 Institute of Power Engineering-Research Institute, Mory 8 Street, 01-330 Warsaw, Poland; agnieszka.zurawska@ien.com.pl; 5 CTH2-Center for Hydrogen Technologies, Institute of Power Engineering, Augustówka 36 Street, 02-981 Warsaw, Poland

**Keywords:** solid oxide fuel cell, solid oxide electrolysis cell, zirconia, manganese oxide, ionic conductivity, thermochemical expansion, oxygen nonstoichiometry, mixed conductor, buffer layer

## Abstract

Manganese-substituted 5 mol.% yttria-stabilized zirconia (5YSZ) was explored as a prospective material for protective interlayers between electrolyte and oxygen electrodes in reversible solid oxide fuel/electrolysis cells. [(ZrO_2_)_0.95_(Y_2_O_3_)_0.05_]_1−*x*_[MnO*_y_*]*_x_* (*x* = 0.05, 0.10 and 0.15) ceramics with cubic fluorite structure were sintered in air at 1600 °C. The characterization included X-ray diffraction (XRD), scanning electron microscopy (SEM)/energy dispersive spectroscopy (EDS), X-ray photoelectron spectroscopy (XPS), thermogravimetry and dilatometry in controlled atmospheres, electrical conductivity measurements, and determination of oxygen-ion transference numbers by the electromotive force (EMF) technique. Mn-substituted 5YSZ solid solutions exhibit variable oxygen nonstoichiometry with manganese cations in a mixed 2+/3+ oxidation state under oxidizing conditions. Substitution by manganese gradually increases the extent of oxygen content variation on thermal/redox cycling, chemical contribution to thermal expansion and dimensional changes on reduction. It also deteriorates oxygen-ionic conductivity and improves *p*-type electronic conductivity under oxidizing conditions, leading to a gradual transformation from predominantly ionic to prevailing electronic transport with increasing *x*. Mn^2+/3+^→Mn^2+^ transformation under reducing atmospheres is accompanied by the suppression of electronic transport and an increase in ionic conductivity. All Mn-substituted 5YSZ ceramics are solid electrolytes under reducing conditions. Prolonged treatments in reducing atmospheres, however, promote microstructural changes at the surface of bulk ceramics and Mn exsolution. Mn-substituted 5YSZ with 0.05 ≤ *x* < 0.10 is considered the most suitable for the interlayer application, due to the best combination of relevant factors, including oxygen content variations, levels of ionic/electronic conductivity and thermochemical expansion.

## 1. Introduction

Solid oxide cells (SOCs) are attractive high-temperature electrochemical systems that offer a solution for the efficient utilization of renewable energy. Solid oxide electrolysis cells (SOECs) can utilize excess electrical energy produced by renewable sources to generate green hydrogen as a fuel or energy storage, while solid oxide fuel cells (SOFCs) may convert chemical energy stored in H_2_ back into electricity with high conversion efficiency [1,2]. SOEC technology is based on the same main components and materials used in SOFCs, but is at a comparatively earlier development stage.

Long-term degradation remains a major obstacle to the introduction of this technology as a practical hydrogen production system. While some lifetime-limiting factors are common with SOFCs, and originate from high operation temperatures, a specific degradation mechanism in SOECs is related to electrolyte deterioration and delamination phenomena at or near electrolyte/anode interface [3,4,5,6,7]. Experimental and modeling results suggest that, under certain conditions, high oxygen pressures can develop in the electrolyte near the oxygen electrode/electrolyte interface (possibly resulting from insufficient electrochemical activity of the electrode materials for the oxygen evolution reaction) [6,7,8,9]. This oxygen pressure build-up may result in the formation of voids at the grain boundaries and intergranular fractures in the surface region of the solid electrolyte, delamination of the oxygen electrode, and the formation of cracks in the composite oxygen electrode [4,5,6,7]. All these factors contribute to the irreversible degradation of SOEC, including an increase in area-specific ohmic/polarization resistance and mechanical failure. Cation interdiffusion was also reported to facilitate the degradation of electrode/electrolyte interface, particularly in the case of oxygen electrodes based on classic (La,Sr)MnO_3−δ_ (LSM) [10,11].

Solid oxide fuel/electrolysis cells tests [3,12,13] demonstrated better tolerance to electrochemical degradation under reversible cycling rather than under continuous electrolysis, possibly by reversing contaminations or electrically driven cation de-mixing and chemical potential gradients across the cell components by orders of magnitude. This raises prospects to halt degradation on reverting from electrolysis to fuel cell mode, and to seek self-healing approaches based on reversible SOEC/SOFC cycles [13]. One possible approach may be based on the introduction of inclusions (in solid electrolyte) or a thin buffer interlayer with oxygen storage ability, preventing oxygen overpressure in electrolysis mode and reverting to oxygen loss in fuel cell mode.

The present work aims at the characterization of manganese- and yttria- co-substituted zirconia as a prospective interlayer material for reversible SOCs. Earlier reports demonstrated that the solubility of manganese oxide MnO*_y_* in ZrO_2_ under oxidizing conditions is limited, 5.4 mol.% [14] or even lower [15] at 1400 °C. Co-substitutions by MnO*_y_* and yttria expand the solid solution formation field; the solubility limit of Mn in ZrO_2_ lattice increases with increasing temperature and Y content [14,16,17,18,19,20,21]. Furthermore, the additions of MnO*_y_* enable the stabilization of cubic fluorite structure at lower yttria contents compared to the binary ZrO_2_-Y_2_O_3_ system [14,17,22]. Hereafter, the conventional abbreviation *n*YSZ is used to refer to *n* mol.% yttria-stabilized zirconia, e.g., 8YSZ corresponds to (ZrO_2_)_0.92_(Y_2_O_3_)_0.08_. Electrical studies reported in literature focused mainly on additions of manganese to 8YSZ [19,20,21,22,23,24,25,26,27,28], with only several publications on additions to 3YSZ [22,29]. Most reports agree that Mn additions (within the solid solubility limits) to 8YSZ result in a decrease in total electrical conductivity under oxidizing conditions, with an increase in activation energy [20,21,23,24,25,26,27,28]. This is accompanied by a gradual increase in *p*-type electronic contribution [22,23,24], although there is a substantial scattering in obtained oxygen-ion transference numbers: from >0.99 [20,21,23] to ~0.2 [24] for similar compositions under similar conditions. Small additions of manganese oxide to 3YSZ were also found to suppress electrical (ionic) conductivity [29], while 3YSZ, with 10–15 mol.% of MnO*_y_*, were claimed to be predominantly electronic conductors [22]. Nonetheless, Mn-substituted 8YSZ was suggested as an intermediate layer between the electrolyte and the oxygen electrode in SOFC/SOEC [21,30]. In particular, the introduction of a 2 mol.% MnO*_y_*-8YSZ interlayer in a model SOEC was demonstrated to suppress the interfacial degradation and delamination of oxygen electrodes by inhibiting Mn diffusion from the LSM electrode into 8YSZ electrolyte [30].

The present work investigates the effect of manganese co-substitution on the properties of YSZ with a lower yttria content compared to conventional 8YSZ. The compositions were formulated as [(ZrO_2_)_0.95_(Y_2_O_3_)_0.05_]_1−*x*_[MnO*_y_*]*_x_*, with *x* varying between 0.05 and 0.15. The cation ratios recalculated for the AO_2_ formula unit are listed in Table 1. Detailed characterization includes structural and microstructural studies, measurements of thermochemical expansion and electrical conductivity in controlled atmospheres, determination of ionic and electronic contributions to the total electrical transport, and assessment or redox behavior by thermogravimetry and conductivity relaxation experiments.

## 2. Materials and Methods

Ceramic samples with composition [(ZrO_2_)_0.95_(Y_2_O_3_)_0.05_]_1−*x*_[MnO_y_]*_x_* (*x* = 0.05, 0.10 and 0.15) were prepared by a solid-state synthesis route employing 5 mol.% yttria partially stabilized zirconia (ZrO_2_)_0.95_(Y_2_O_3_)_0.05_ (Innovnano, Portugal, d_50_ = 500 nm) and manganese (IV) oxide MnO_2_ (Alfa Aesar, ThermoFisher, Kandel, Germany, 99.9% purity, 325 mesh powder, particles agglomerates ≤ 10 µm) as starting reagents. The reagent powders were weighed and mixed in appropriate proportions and then balled-milled with ethanol for 4 h at 150 rpm (Retsch S1 planetary mill, nylon containers, Tosoh tetragonal zirconia milling media). After milling and drying, the powders were subjected to consecutive calcination steps in air at 800 °C, 1000 °C, and three times at 1200 °C, 5 h each step, with intermediate regrinding. Furthermore, after repeated ball-milling, the powders were compacted into disk-shaped samples (ф 18 mm, thickness ~1.5 mm) by uniaxial pressing (F = 10 kN) and sintered in air at 1600 °C for 10 h. Sintering was done using alumina plates as supports covered with alumina cups; each sample was placed on a bed of powder, and also covered with a powder of identical cation composition to act as a buffer against possible high-temperature MnO*_y_* losses [18,31].

Sintered ceramics samples were polished and cut into rectangular bars for electrical and dilatometric measurements. The density of prepared ceramics was calculated from the geometric dimensions and mass of polished samples. Powdered samples for X-ray diffraction (XRD), X-ray photoelectron spectroscopy (XPS) and thermogravimetric analysis (TGA) studies were prepared by grinding sintered ceramics in a mortar.

XRD patterns were recorded at room temperature using a PANalytical X’Pert PRO MRD diffractometer (CuK_α_ radiation, PANalytical, Almelo, Netherlands) in 2Θ range of 20–80° for phase analysis and 10–120° for lattice parameter calculations. Unit cell parameters were refined in FullProf software (profile matching method, WinPLOTR version Sept. 2018, CDIFX UMR6226 Rennes, ILL Grenoble, France). Microstructural characterization was performed by scanning electron microscopy (SEM, Hitachi SU-70, Tokyo, Japan) coupled with energy dispersive spectroscopy (EDS, Bruker Quantax 400 detector, Berlin, Germany). XPS studies were performed on a PHI Versa Probe II spectrometer (Physical Electronics, Minneapolis, MN, USA) with monochromatic X-ray Al K_α_ radiation (100 µm, 100 W, 20 kV, 1486.6 eV) and a dual-beam charge neutralizer. The instrument work function of the spectrometer was calibrated using Cu 2*p*_3/2_ (932.7 eV), Ag 3*d*_5/2_ (368.2 eV) and Au 4*f*_7/2_ (84.0 eV) photoelectron lines. The collected XPS spectra were analyzed using PHI SmartSoft software (version 9.6.0, PHI, Minneapolis, MN, USA) and processed with MultiPak. Charge referencing was done against the adventitious carbon C 1*s* signal (284.8 eV). Recorded spectra were fitted using Gauss-Lorentz curves.

Dilatometric measurements were done at 25–1100 °C using a vertical Linseis L75 instrument in flowing air or 10% H_2_-N_2_ gas mixture with a constant heating/cooling rate of 3 °C/min. TGA was performed at 25–1100 °C using Setaram Setsys 16/18 instrument (sensitivity 0.4 µg, alumina crucibles, initial sample weight ~0.54 g) on heating/cooling at 2 °C/min in flowing air or 10% H_2_-N_2_ gas mixture or isothermally as a function of time on cycling between oxidizing and reducing atmospheres. All thermogravimetric data were corrected for buoyancy effects by subtracting the corresponding baselines recorded under identical conditions using dense inert ceramic samples of a similar volume.

Total electrical conductivity (σ) was measured employing AC impedance spectroscopy (Keysight E4284A precision LCR meter, Santa Rosa, CA, USA, frequency range 20 Hz–2 MHz) and disk- or bar-shaped ceramic samples with applied porous Pt electrodes (Heraeus CL-11-5349 platinum paste, Hanau, Germany). The measurements were done in air in the temperature range of 500–1000 °C or isothermally at 700–900 °C as function of oxygen partial pressure using flowing O_2_ + N_2_ and H_2_ + H_2_O + N_2_ gas mixtures. The average oxygen-ion transference numbers ($\bar{t_{O}}$) under air/O_2_ and air/(10% H_2_-N_2_) gradients were determined at 700–900 °C by the electromotive force (EMF) technique modified to take the electrode polarization into account (Gorelov’s modification) (see References [32,33,34] for details). Only gas-tight samples were used for the EMF measurements. The samples were hermetically sealed onto yttria-stabilized zirconia (YSZ) tubular measuring cell using G018-281 glass (Schott AG, Mainz, Germany) in a form of tape-casted glass rings and a glass paste (a mixture of glass powder with terpineol and ethyl cellulose).

In all experiments, the flow rates of gases were controlled using Bronkhorst mass-flow controllers (Ruurlo, The Netherlands). The oxygen partial pressure p(O_2_) in the gas mixtures was monitored by YSZ solid-electrolyte sensors (homemade, YSZ tubes from Tosoh, Tokyo, Japan). The representative p(O_2_) value in the 10%H_2_-N_2_ gas mixture corresponded to ~10^−21^ atm at 900 °C. Note that p(O_2_) in this kind of gas mixtures is defined by the p(H_2_O)/p(H_2_) ratio and by the temperature-dependent equilibrium constant of hydrogen oxidation reaction, and therefore varies with temperature.

## 3. Results

### 3.1. Phase Composition and Crystal Structure

**Figure 1:**
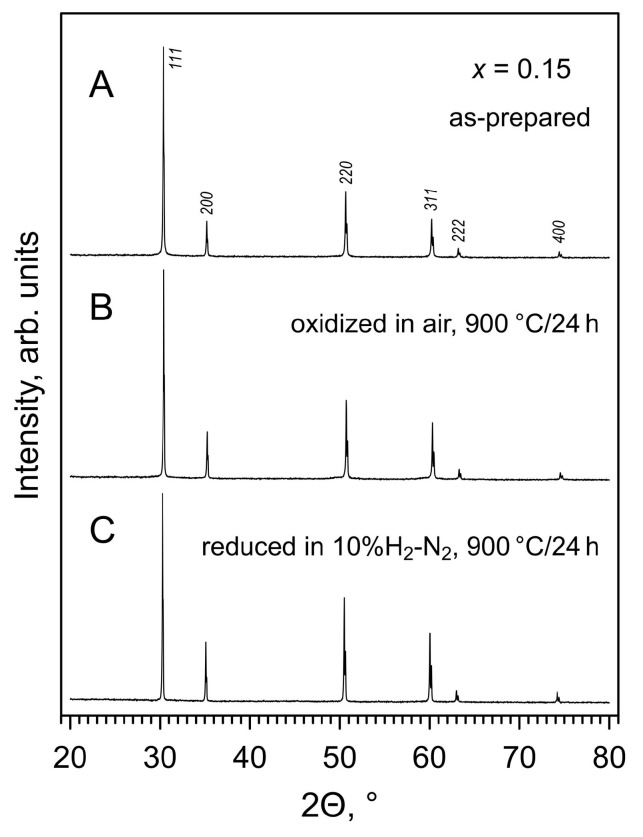
X-ray diffraction (XRD) patterns of crashed [(ZrO_2_)_0.95_(Y_2_O_3_)_0.05_]_0.85_[MnO*_y_*]_0.15_ ceramics: (**A**) as-prepared; (**B**) oxidized in air at 900 °C for 24 h; and (**C**) reduced in 10% H_2_-N_2_ flow at 900 °C for 24 h. XRD reflections are indexed in the space group *Fm*$\bar{3}$*m*.

XRD analysis confirmed that (ZrO_2_)_0.95_(Y_2_O_3_)_0.05_, either as-delivered powder used for synthesis or ceramic samples sintered in air at 1600 °C for 10 h, is partially-stabilized zirconia with tetragonal structure and a minor fraction of monoclinic phase. Additions of manganese oxide resulted in the stabilization of the fluorite cubic structure (space group *Fm*$\bar{3}$*m*). All prepared [(ZrO_2_)_0.95_(Y_2_O_3_)_0.05_]_1−*x*_[MnO*_y_*]*_x_* (*x* = 0.05, 0.10 and 0.15) ceramics were single-phase, with no evidence of impurity phases detectable by XRD. An example of XRD pattern of as-prepared ceramics is given in Figure 1A.

**Figure 2:**
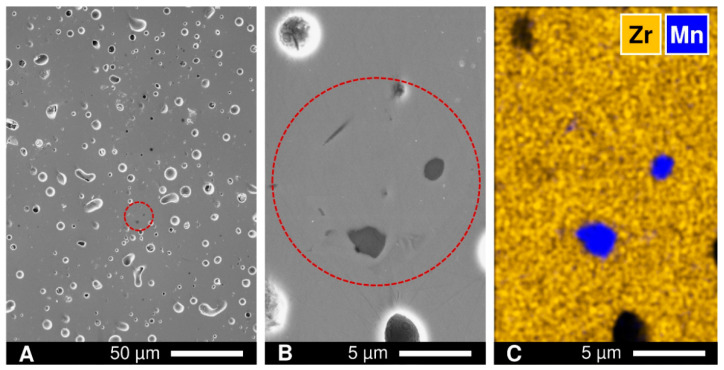
(**A**) Scanning electron microscopy (SEM) image of polished as-prepared [(ZrO_2_)_0.95_(Y_2_O_3_)_0.05_]_0.85_[MnO*_y_*]_0.15_ ceramics; (**B**) magnified image of selected area; and (**C**) corresponding energy dispersive spectroscopy (EDS) elemental mapping.

Polished as-prepared ceramic samples were further inspected by SEM/EDS. Microscopic studies revealed the presence of occasional isolated inclusions of MnO*_y_* with sizes ≤ 2 µm, mainly in the *x* = 0.15 samples (Figure 2). The concentration of inclusions was low (<0.2 vol.% for *x* = 0.15, as roughly estimated from the SEM images), so that its influence on the properties studied in this manuscript is considered negligible.

The analysis of available literature data suggests that the solubility of manganese oxide in (Zr,Y)O_2−δ_ is limited, but varies with yttrium content and temperature. In particular, the equilibrium solubility limits of MnO*_y_* at 1400 °C in air were reported to be 7.6, 10.3 and 11–12 mol.% in 3, 6 and 8 mol.% yttria-substituted zirconia, respectively [14,17,18,19]. Increasing temperature extends the solubility of MnO*_y_* in (ZrO_2_)_0.92_(Y_2_O_3_)_0.08_ from ~5 mol.% at 1000 °C to 15–18 mol.% at 1500 °C [19,21]. Thus, it can be assumed that the manganese contents in fluorite-type ceramics prepared in the present work, particularly in samples with *x* = 0.15, are not equilibrium, but quenched from sintering temperature. This is also supported by the previous results on the processing of the *x* = 0.15 ceramics at 1400–1600 °C [35], showing that the concentration and size of MnO*_y_* precipitates increase with a reduction in time and the temperature of firing.

**Figure 3:**
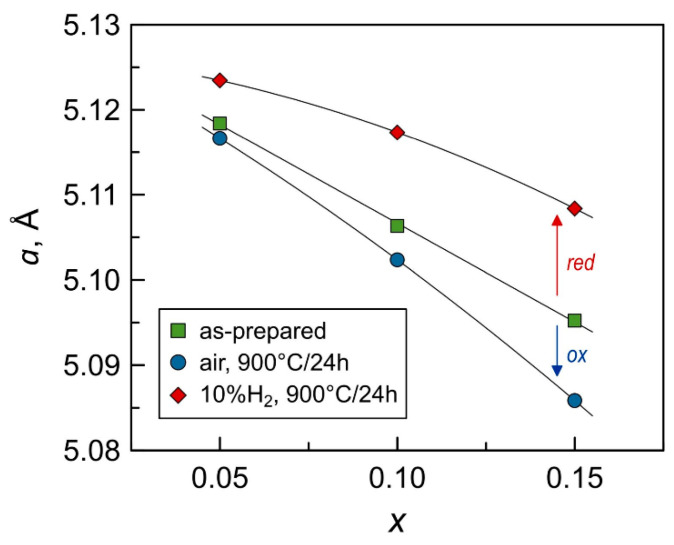
Lattice parameters of fluorite [(ZrO_2_)_0.95_(Y_2_O_3_)_0.05_]_1−*x*_[MnO*_y_*]*_x_* solid solutions as function of manganese content: as prepared, after oxidation in air at 900 °C for 24 h, and after reduction in 10% H_2_-N_2_ flow at 900 °C for 24 h.

The lattice parameter of as-prepared fluorite-type solid solutions decreases linearly with increasing manganese content (Table 1 and Figure 3). The ionic radius of zirconium cations in an 8-fold coordination in the cubic fluorite lattice is 0.84 Å [36]. Thus, a decrease in the lattice parameter with *x* can reasonably be attributed to the incorporation of manganese cations in a lower coordination (*r*^VI^(${\mathrm{Mn}}_{LS}^{3+})$ = 0.58 Å, *r*^VI^(${\mathrm{Mn}}_{HS}^{3+})$ = 0.65 Å or *r*^VI^(${\mathrm{Mn}}_{LS}^{2+})$ = 0.67 Å [36]) into the zirconia lattice combined with a simultaneous moderate decrease in the concentration of large yttrium cations (*r*^VIII^(Y^3+^) = 1.02 Å [36]).

The density of prepared ceramics was 90–94% of theoretical (Table 1). The grain size was in the range of 2–20 µm for *x* = 0.05 and 3–80 µm for *x* = 0.10–0.15. Additions of manganese resulted in some improvement in sinterability and grain growth, in agreement with literature reports [28,37,38], probably due to the relatively high diffusivity of Mn species in cubic zirconia [39].

### 3.2. Mn Oxidation State and Oxygen Nonstoichiometry in Air

**Figure 4:**
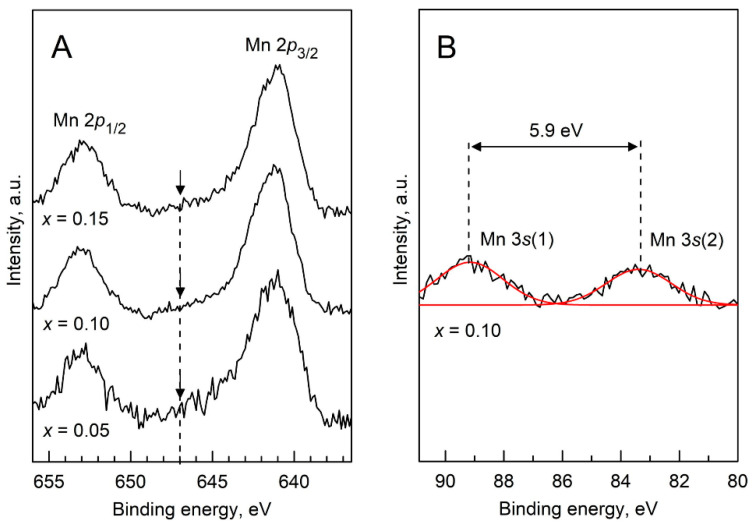
(**A**) X-ray photoelectron spectroscopy (XPS) spectra of [(ZrO_2_)_0.95_(Y_2_O_3_)_0.05_]_1−*x*_[MnO*_y_*]*_x_* ceramics in the Mn 2*p* core level region; and (**B**) Representative fitting of the Mn 3*s* multiplet for the *x* = 0.10 sample. The arrows in (**A**) indicate the position of a satellite peak at a binding energy (BE) of ~647 eV. The spectra are shown with a subtracted background.

The XPS spectra were collected on as-sintered crashed ceramic samples. The binding energy (BE) values of the Zr 3*d*_3/2_ and Y 3*d*_3/2_ signals were found at 158.4–158.6 and 183.8–184.0 eV, respectively, in agreement with the literature data on cubic and tetragonal YSZ [40] and Mn-doped YSZ systems [41,42]. The Mn 2*p* core-level spectra for all compositions are shown in Figure 4A; the corresponding BE values are listed in Table 2. The BEs of the Mn 2*p*_1/2_ and Mn 2*p*_3/2_ signals are given by the positions of the maxima of the main peaks. The asymmetric Mn 2*p*_3/2_ main peak is located at 641.0–641.4 eV with a 2*p*_3/2_–2*p*_1/2_ splitting of 11.7–12.0 eV. These values are closer to the BE of Mn 2*p*_3/2_ peaks reported for MnO (640.6 eV), Mn_3_O_4_ (641.4 eV) and Mn_2_O_3_ (641.9 eV) binary oxides rather than for MnO_2_ (642.2 eV) [43]. Therefore, Mn cations in 2+ and 3+ oxidation states are expected to prevail for all compositions, although a minor presence of Mn^4+^ ions cannot be discarded based on the obtained XPS data. In addition, a satellite peak at a BE of ~647 eV (full width at half maximum (FWHM) = 3.5 eV) was evidenced in all the Mn 2*p* spectra (Figure 4A). This feature is characteristic of MnO [44] and has not been reported for neither Mn_2_O_3_ nor MnO_2_, thus further suggesting the presence of Mn^2+^ species in the samples. A slightly higher intensity of this satellite feature for *x* = 0.05 seems to imply a lower average oxidation state of Mn in this sample. A minor decrease in FWHM of the Mn2*p*_3/2_ peak from 3.3 eV (*x* = 0.05) to 3.0–3.1 eV (*x* = 0.10 and 0.15) is also likely to be related to a lower concentration of Mn^2+^ in the samples with a higher Mn content.

Analysis of the Mn 3*s* core level spectrum is more useful to assess the oxidation state of Mn. A representative fitting of the Mn 3*s* multiplet for the sample with *x* = 0.10 is included in Figure 4B. The spectra exhibited two components with the splitting magnitude being related to the oxidation state of the Mn ions: typically 6.5–5.7 eV for Mn^2+^ in MnO, 5.5–5.2 eV for Mn^3+^ in Mn_2_O_3_, and 4.7–4.5 eV for Mn^4+^ in MnO_2_ [45,46]. The measured Δ(3*s*(2)−3*s*(1)) values (Table 2) ranged from 6.4 eV (*x* = 0.05) to 5.9 eV (*x* = 0.10), thereby supporting a mean Mn oxidation state lower than 3+ in all the samples, as well as a higher concentration of Mn^2+^ species in the *x* = 0.05 sample.

**Figure 5:**
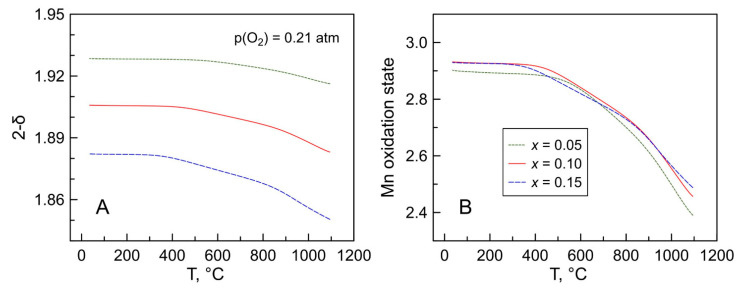
Temperature dependence of: (**A**) oxygen nonstoichiometry per Zr_1−*a*−*b*_Y*_a_*Mn*_b_*O_2−δ_ formula unit; and (**B**) average Mn oxidation state in [(ZrO_2_)_0.95_(Y_2_O_3_)_0.05_]_1−*x*_[MnO*_y_*]*_x_* ceramics in air calculated from the TGA data (cooling regime).

TGA was employed to determine the absolute values of oxygen nonstoichiometry and average manganese oxidation state in Mn-substituted 5YSZ in air (Figure 5). The TGA data were obtained on temperature cycling in air followed by the isothermal reduction of the samples in 10% H_2_-N_2_ flow at 900 °C until constant weight (see discussion below). The calculations were done assuming that all manganese in reduced samples is in a 2+ oxidation state. This assumption is consistent with redox changes in the Mn-O system [47], and also with the evidence by electron paramagnetic resonance (EPR) studies of manganese-doped zirconia [48] and YSZ [49,50], and the results of thermodynamic modeling of the Zr-Y-Mn-O system [51].

The TGA results demonstrated that Mn-substituted 5YSZ ceramics exhibit variable oxygen content on temperature cycling in air above 300–500 °C (depending on composition) associated with reversible reduction/oxidation of manganese cations

(1) $$Mn^{3+}+\frac{1}{2}O^{2-}\overset{T↑}{\underset{T↓}{⇄}}Mn^{2+}+\frac{1}{4}O_{2},$$

or, using Kroger-Vink notation:

(2) $$M{n^{\prime }}_{Zr}+\frac{1}{2}O_{O}^{\times }\overset{T↑}{\underset{T↓}{⇄}}M{n^{\prime \prime }}_{Zr}+\frac{1}{2}V_{O}^{••}+\frac{1}{4}O_{2}$$

The calculated average oxidation state of Mn cations in the studied materials in air is below 3+ (Figure 5B), in accordance with the XPS results. The mean Mn valence is nearly independent of the total manganese content varying in a narrow range 2.61–2.66 at 900 °C and 2.90–2.93 after cooling down to room temperature, although the *x* = 0.05 ceramics showed a tendency to a slightly higher relative fraction of Mn^2+^ compared to other compositions. These results are in excellent agreement with available literature reports, showing a mixed 2+/3+ oxidation state of manganese cation in Mn-doped YSZ. In particular, the results of EPR and optical absorption spectroscopy studies of 9.5YSZ single crystals containing impurity levels of Mn (≤0.1 wt.%) revealed that the fraction of bivalent manganese, [Mn^2+^]/[Mn]_total_, in the samples equilibrated with air at 800 °C is close to 45–50% [49,50,52,53]. Mixed Mn^2+/3+^ oxidation state in manganese-doped 8YSZ ceramics was demonstrated by EPR [48] and XANES (X-ray Absorption Spectroscopy in the Near Edge region) [16] studies and supported by the thermodynamic modeling of this system [51]. Appel et al. [54] performed EELS (Electron Energy Loss Spectroscopy) studies of Mn-doped 7.5YSZ ceramics, and also concluded that Mn cations are in a mixed 2+/3+ state, and that the average Mn oxidation state does not change with manganese content in the studied compositional range (2–10 mol.% of MnO*_y_*). The presence of Mn^4+^ in Mn-doped YSZ was ruled out by EPR measurements, even under oxidizing conditions [48,50].

The substitution of Zr^4+^ by lower-valence Mn^3+/2+^ generates oxygen vacancies in the fluorite lattice. The electroneutrality condition for Mn-substituted YSZ is given by

(3) $$2[M{n^{\prime \prime }}_{Zr}]+[M{n^{\prime }}_{Zr}]+[{Y^{\prime }}_{Zr}]=2[V_{O}^{••}]=2\delta$$

The increase in manganese content results in a gradual increase in the concentration of oxygen vacancies and also in the extent of oxygen nonstoichiometry variations with temperature (Figure 5A). A specific feature observed in the thermogravimetric curves, and therefore in temperature dependencies of calculated parameters, is a change in slope at 820–880 °C, probably corresponding to a limiting range for partial relaxation of quenched-in conditions. The exact reason for this is under question; possible factors may include a change of limiting stage of redox re-equilibration process (surface exchange ↔ bulk diffusion) or order-disorder processes in the fluorite lattice.

### 3.3. Electrical Transport Properties under Oxidizing Conditions

**Figure 6:**
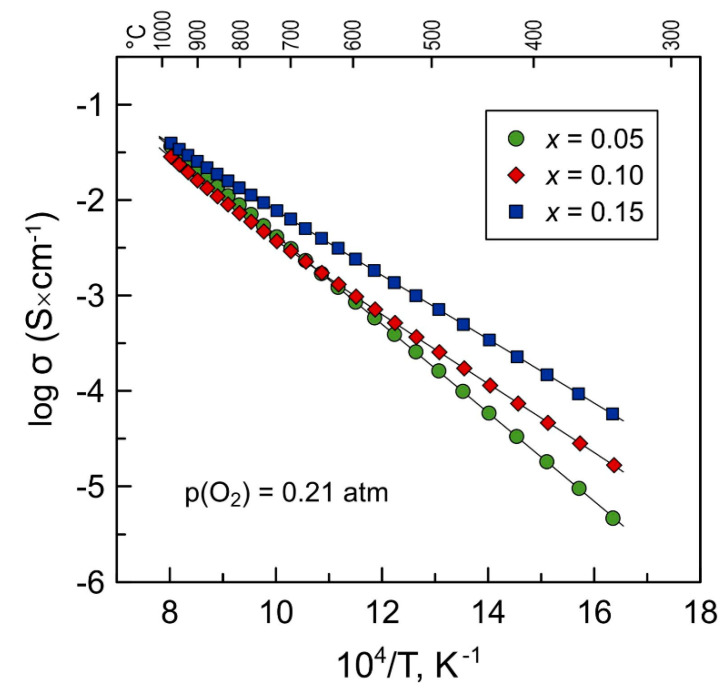
Temperature dependence of total electrical conductivity of as-prepared Mn-substituted 5YSZ ceramics in air.

All prepared Mn-substituted 5YSZ ceramics exhibit semiconducting behavior: electrical conductivity increases on heating. As-prepared ceramic materials show a comparable level of conductivity in the high-temperature range in air (Figure 6). Increasing the Mn content from *x* = 0.05 to *x* = 0.10 results in a moderate reduction in σ, while further substitution results in an enhancement in electrical transport. At the same time, substitution by manganese is accompanied by a gradual decrease in the activation energy of electrical conductivity (Table 3). As a result, the electrical conductivity of the studied materials increases with the Mn concentration in the fluorite lattice at temperatures below ~650 °C. 

**Figure 7:**
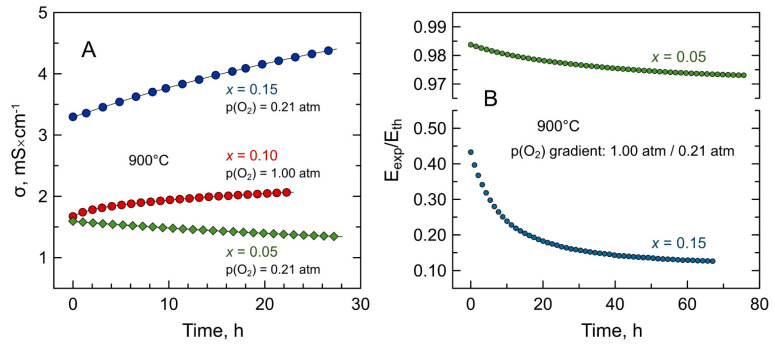
Examples of relaxation of: (**A**) electrical conductivity of as-prepared Mn-substituted 5YSZ ceramics under oxidizing conditions at 900 °C; and (**B**) observed E_exp_/E_th_ values of the samples in air/O_2_ concentration cells at 900 °C.

Isothermal electrical measurements at 900 °C revealed, however, that all the Mn-substituted 5YSZ exhibited a quite slow relaxation of electrical conductivity with time under oxidizing conditions. This was observed even for as-prepared samples in atmospheric air: the conductivity of *x* = 0.10 and *x* = 0.15 samples tended to increase with time, while the sample with *x* = 0.05 showed the opposite behavior (Figure 7A). Thermogravimetric studies showed that this is accompanied by a slow weight gain (i.e., oxygen uptake) for all compositions.

**Figure 8:**
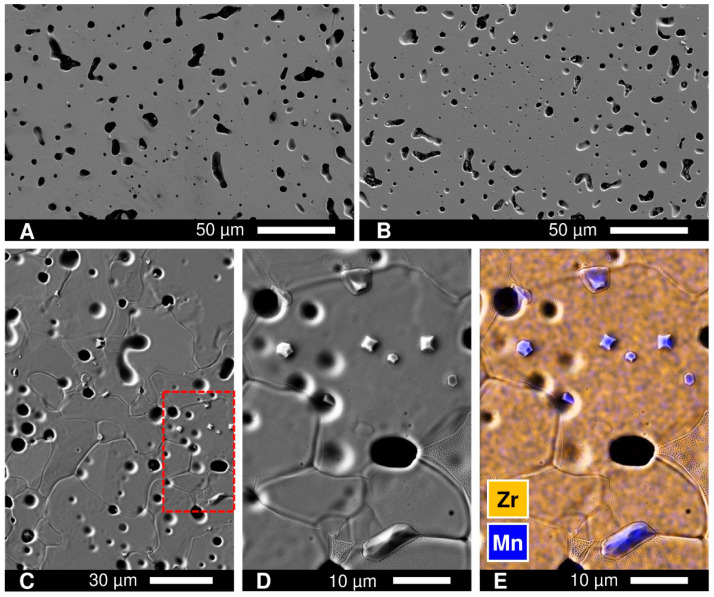
SEM images of the *x* = 0.15 ceramics: (**A**) as-prepared polished; (**B**) polished and annealed in air at 900 °C for 360 h; (**C**) polished and heated in air to 1400 °C with immediate cooling (heating/cooling at 5 °C/min); (**D**) magnified image of selected area in (**C**); and (**E**) magnified image with overlaid EDS elemental mapping.

Since the Mn content in prepared materials at temperatures ≤ 1000 °C is expected to be non-equilibrium (quenched from sintering conditions), a possible explanation for the slow transient processes occurring even in air at 900 °C could be very sluggish exsolution of the excess of Mn from the fluorite lattice; however, one could not find microstructural changes supporting this. No evidence of Mn segregation on the surface of polished samples was observed by SEM/EDS, even after prolonged annealing at 900 °C for 360 h (Figure 8). On the contrary, even a short treatment at the higher temperature, 1400 °C, resulted in manganese exsolution from the bulk of ceramics and accumulation of MnO*_y_* grains on the surface (Figure 8). This suggests that cation diffusivity and the phase re-equilibration at ≤900 °C are very slow, in agreement with some indications in literature [19], and are unlikely to be responsible for the observed relaxation processes.

The structural studies also do not support the possible exsolution of Mn. A significant decrease in manganese concentration in the fluorite lattice would result in a lattice expansion and the onset of a Mn-rich phase. On the contrary, it was found that while all materials remain single-phase solid solutions (Figure 1B), annealing in air at 900 °C results in a contraction of the fluorite unit cell (Figure 3). This implies that slow oxygen uptake at 900 °C is associated with Mn^2+^ → Mn^3+^ oxidation, and lattice shrinkage is caused by a corresponding decrease in the average size of manganese cations (e.g., *r*^VI^(${\mathrm{Mn}}_{HS}^{3+})$ = 0.65 Å and *r*^VI^(${\mathrm{Mn}}_{HS}^{2+})$ = 0.83 Å [36]).

A slow relaxation process at 900 °C was also observed in the course of ionic transference numbers determination by the EMF method. Figure 7B illustrates the drift of E_exp_/E_th_ ratio with time for air/Pt/(Mn-substituted YSZ)/Pt/O_2_ concentration cells. E_exp_ and E_th_ designate experimentally measured open-circuit voltage of the cell and theoretical voltage defined by the Nernst equation, respectively. In the classic EMF method, the E_exp_/E_th_ ratio gives an average ionic transference number in a given p(O_2_) range. Gorelov’s modification of the EMF method [32,55,56] was employed in the present work to account for the non-negligible polarization of cell electrodes resulting in underestimation of ionic transference numbers. As shown in Figure 7B, E_exp_/E_th_ values decreased slowly with time and required over 70 h at 900 °C to stabilize. The measured E_exp_/E_th_ ratio and the average oxygen-ion transference numbers at the beginning of the experiment (immediately after sealing the sample at 950 °C and cooling down to 900 °C) and after ≥70 h of sample equilibration are summarized in Table 4. For all Mn-substituted 5YSZ ceramics, E_exp_/E_th_ and $\bar{t_{O}}$ values decreased after the relaxation process implying a decline in ionic contribution and/or an enhancement of electronic contribution to total electrical transport. The extent of these changes is correlated with the nominal Mn concentration. In agreement with electrical conductivity measurements (Figure 7A), the ohmic resistance of the *x* = 0.05 sample increased during the relaxation process, while *x* = 0.10 and 0.15 samples exhibited a decrease in resistance with time (Table 4).

**Figure 9:**
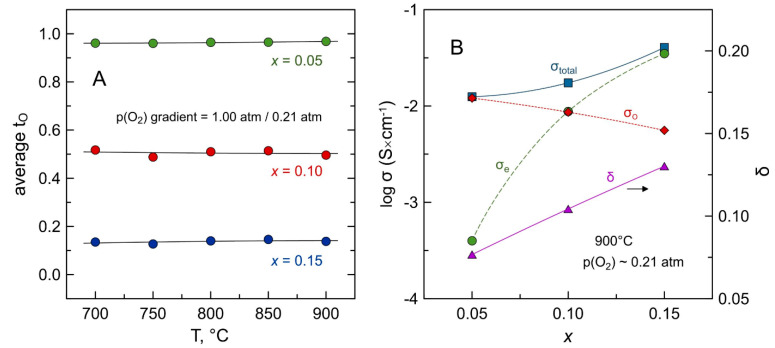
(**A**) Temperature dependence of average oxygen-ion transference numbers of Mn-substituted 5YSZ ceramics under air/O_2_ gradient; and (**B**) total and partial ionic and electronic conductivities and oxygen deficiency (per AO_2_ formula unit) at 900 °C in air as function of Mn content. The values of σ_O_ and σ_e_ are estimated from the data on total conductivity in air and average t_O_ under air/O_2_ gradient. All values were obtained after isothermal relaxation at 900 °C (≥70 h for t_O_; ≥25 h for σ; ≥10 h for δ).

Figure 9A shows the temperature dependence of the average oxygen-ion transference numbers of Mn-substituted 5YSZ under air/oxygen gradient after equilibration at 900 °C. All compositions demonstrated negligible dependence of $\bar{t_{O}}$ on temperature at 700–900 °C. Substitution by manganese leads to a gradual transformation from a predominantly ionic conductor (*x* = 0.05) to a mixed conductor with similar contributions of ionic and electronic transport (*x* = 0.10), and then to a material with prevailing electronic conduction (*x* = 0.15) under oxidizing conditions close to air.

Calculations of partial contributions to total conductivity demonstrated that increasing Mn concentration in the fluorite lattice results in the gradual enhancement of electronic transport but simultaneously suppresses ionic conduction (Figure 9B). Yttria-doped zirconia is known to exhibit very low *p*-type electronic conductivity under oxidizing conditions. For instance, the electronic conductivity of 8YSZ in air at 900 °C was reported to be as low as ~10^−5^ S × cm^−1^ [57]. Additions of manganese lead to an increase in electronic conductivity by orders of magnitude (Figure 9B). Similar observations were previously reported for Mn-doped 3YSZ [22] and 8YSZ [19,21,22,23,24] within the manganese solubility ranges.

Oxygen-ionic conductivity of Mn-substituted 5YSZ at 900 °C decreases twice with increasing Mn content (from 1.2 mS × cm^−1^ for *x* = 0.05 to 0.56 mS × cm^−1^ for *x* = 0.15), despite an increase in the concentration of oxygen vacancies (Figure 9B), and is lower compared to 8YSZ (δ = 0.074, σ_O_ = 10 mS × cm^−1^ at 900 °C [58]). It is known that zirconia-based solid electrolytes exhibit the highest oxygen-ionic conductivity when the concentration of acceptor-type dopant (alkaline-earth or rare-earth cation) is close to the minimum required for the stabilization of cubic fluorite structure [59,60]. For the (ZrO_2_)_1−*x*_(Y_2_O_3_)*_x_* system, this corresponds to *x* = 0.08–0.10 [60,61]. Further substitution results in a decline in the ionic conductivity, mainly due to defect association that causes a decrease in the mobility of ionic charge carriers. As for other zirconia-based systems, a decrease in σ_O_ with manganese content in the studied range of solid solution can be assigned, most likely, to coulombic interaction between the point defects, manganese cations ${\mathrm{Mn}}_{\mathrm{Zr}}^{\prime \prime }$ and ${\mathrm{Mn}}_{\mathrm{Zr}}^{\prime }$ and oxygen vacancies $V_{O}^{••}$, and formation of complex defect associates. Previously, Appel et al. [54,62] suggested the association of manganese ions and oxygen vacancies and the formation of ordered microdomains in Mn-doped 7.5YSZ (5–10 mol.% of MnO*_y_*), based on EELS and electron diffraction studies. Kawada et al. [19] found that the ionic conductivity in Mn-substituted 8YSZ in air increases with manganese substitution of up to 4 mol.% of MnO_y_, and declines on further substitution; they also attributed it to the defect association at high Mn substitution levels. An additional factor possibly contributing to the decrease in ionic transport with Mn substitution is the strain caused by lattice shrinkage, which may result in an increase in the migration barrier for oxygen ion diffusion [24].

**Figure 10:**
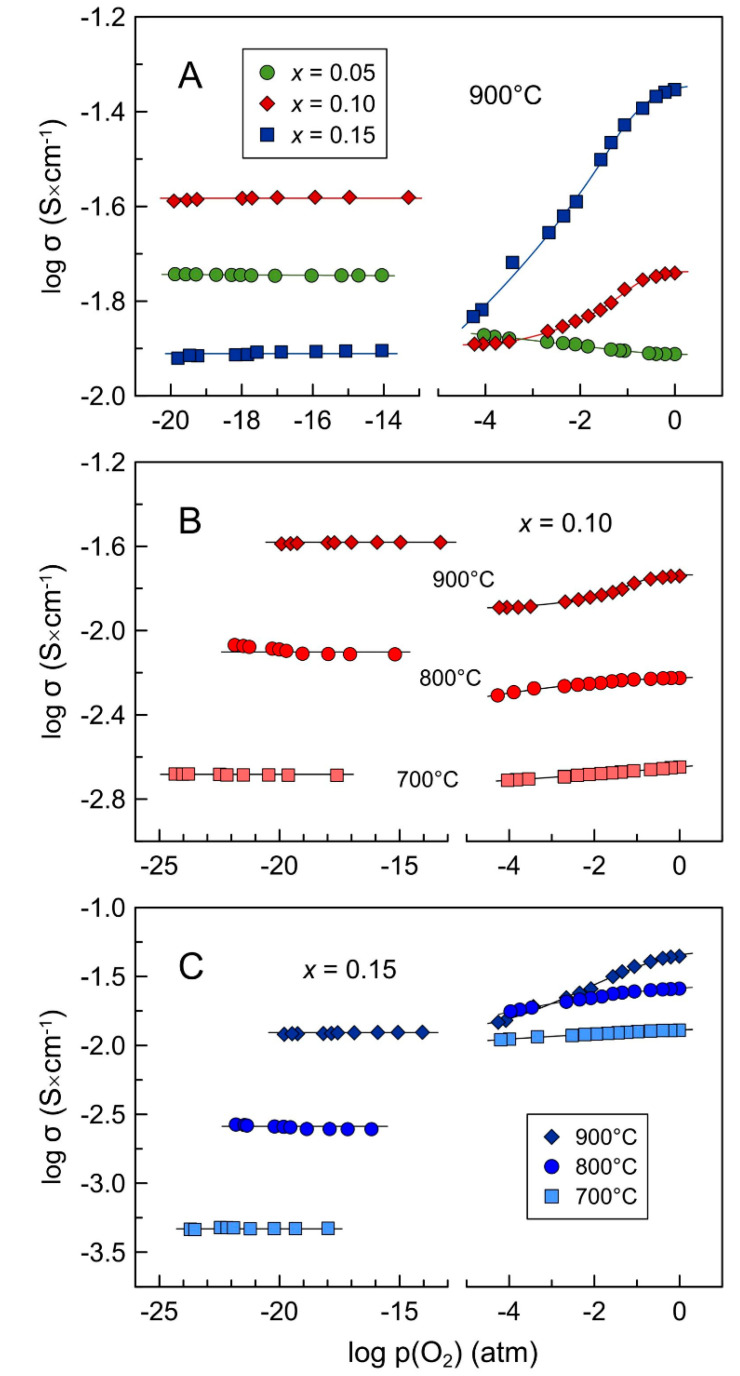
Oxygen partial pressure dependence of electrical conductivity of (**A**) Mn-substituted 5YSZ ceramics at 900 °C; (**B**) *x* = 0.10 ceramics at 700–900 °C; and (**C**) *x* = 0.15 ceramics at 700–900 °C.

The behavior of total electrical conductivity vs oxygen partial pressure under oxidizing conditions, in the p(O_2_) range between 10^−5^ and 1.0 atm (Figure 10), is an interplay between ionic and electronic contributions to electrical transport, and is defined by dominating charge carriers. For all compositions, the defect equilibrium is governed by

(4) $$M{n^{\prime \prime }}_{Zr}+\frac{1}{2}V_{O}^{••}+\frac{1}{4}O_{2}\overset{pO_{2}↑}{\underset{pO_{2}↓}{⇄}}M{n^{\prime }}_{Zr}+\frac{1}{2}O_{O}^{\times },$$

or

(5) $$V_{O}^{••}+\frac{1}{2}O_{2}\overset{pO_{2}↑}{\underset{pO_{2}↓}{⇄}}O_{O}^{\times }+2h^{•},$$

where $h^{•}$ is the electron-hole, in combination with the electroneutrality condition given by Equation (3). The contribution of electronic transport to the total conductivity in the *x* = 0.05 ceramics is only a few percent in air. As a result, the total conductivity of this material shows a moderate increase with reducing p(O_2_) due to an increase in the oxygen vacancy concentration, and therefore, ionic conductivity. Similar behavior was reported for 3YSZ with 3.0–7.6 mol.% MnO*_y_* under oxidizing conditions at 1000–1100 °C [29]. On the contrary, electronic conductivity prevails in the *x* = 0.15 sample in air, when electron holes should become the dominant charge carriers. Note that the contribution of polarons by transfer between Mn^3+^ and Mn^2+^ is less likely, due to the diluted state of manganese cations in the fluorite lattice. Reducing p(O_2_) is accompanied by a decrease in $\left[h^{•}\right]$, *p*-type electronic transport and the total conductivity. This coincides with the dependencies observed for 8YSZ doped by 6–10 mol.% MnO_y_ under oxidizing conditions [20,24]. The *x* = 0.10 solid solution with the electronic transference number close to 0.5 in air exhibits smother variations of σ_total_ with oxygen partial pressure compared to the *x* = 0.15 ceramics. For all compositions, the dependencies of defect concentration and electrical conductivity become weaker at lower temperatures.

Note also that the obtained σ-p(O_2_) dependencies under oxidizing conditions agree well with the trends in variation of conductivity and transference numbers during the initial relaxation of as-prepared samples at 900 °C (Figure 7). Slow oxygen uptake with time is associated with the oxidation of manganese cations, generation of electron-holes and elimination of oxygen vacancies, leading to a decrease in ionic conductivity and an increase in hole transport. Considering the non-negligible and nearly reversible changes of the oxygen content on temperature cycling (Figure 5), a slow oxygen exchange with the gas phase cannot be a reason for a prolonged equilibration of the samples at 900 °C. The slow processes of defect association and clustering seem to be a more likely explanation, although more detailed studies are required to find the exact reasons.

### 3.4. Cycling between Oxidizing and Reducing Conditions

**Figure 11:**
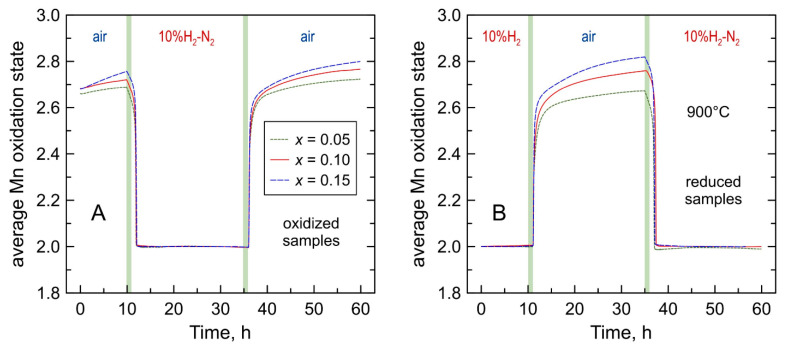
Variations of average manganese oxidation state in Mn-substituted 5YSZ on redox cycling at 900 °C: (**A**) oxidized samples in ox → red → ox cycles; and (**B**) reduced samples (24 h in 10% H_2_-N_2_ flow at 900 °C) in red → ox → red cycle.

Thermogravimetric studies showed that all Mn-substituted 5YSZ powdered samples demonstrate an apparently reversible behavior on cycling between air and 10% H_2_-N_2_ atmospheres at 900 °C (Figure 11A). The samples show a slow oxygen uptake with time in air, but nearly instantly lose oxygen upon switching to a reducing atmosphere, and then exhibit a constant weight suggesting a full reduction of manganese cations to a 2+ oxidation state. Switching back to oxidizing atmosphere results in a rapid oxidation to a state corresponding to the mean manganese valence of 2.55–2.60. This is followed, again, by a slow further oxidation.

**Figure 12:**
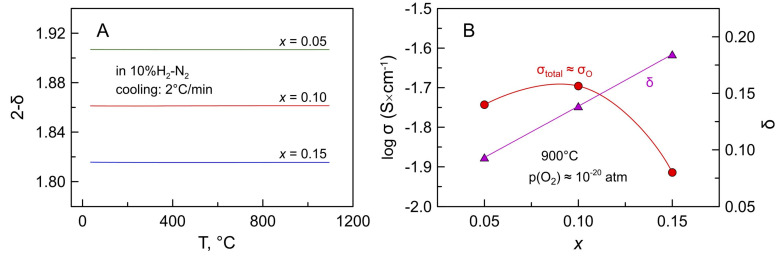
(**A**) Oxygen nonstoichiometry (per AO_2_ formula unit) of reduced Mn-substituted 5YSZ on cooling in reducing atmosphere; and (**B**) Total/ionic conductivity and oxygen deficiency of reduced Mn-substituted 5YSZ at 900 °C as a function of Mn content.

XRD analysis confirmed that all samples remain single-phase solid solutions with cubic fluorite structure after annealing in 10% H_2_-N_2_ flow for 24 h at 900 °C (Figure 1C). The reduction leads to an expansion of the fluorite lattice (Figure 3) caused by an increase in the average ionic radius of manganese cation on Mn^3+/2+^→Mn^2+^ transformation. Thermogravimetric studies also confirmed that manganese oxidation state, and therefore the oxygen nonstoichiometry remains constant on thermal cycling in 10% H_2_-N_2_ atmosphere (Figure 12A), and that the reduced samples exhibit a reversible behavior on isothermal cycling between reducing and oxidizing atmospheres (Figure 11B).

Electrical studies demonstrated that reduced Mn-substituted 5YSZ ceramics exhibit essentially p(O_2_)-independent electrical conductivity under reducing conditions (Figure 10). Reduction at 900 °C resulted in an enhancement of total conductivity of *x* = 0.05–0.10 ceramics compared to oxidizing conditions; the degree of this enhancement diminishes with decreasing temperature. On the contrary, the *x* = 0.15 samples showed a drop in electrical conductivity after reduction at 900 °C, and the extent of this drop became larger with decreasing temperature. Table 5 summarizes the results of measurements of average oxygen-ion transference numbers using air/(10% H_2_-N_2_) concentration cells. The results suggest that all Mn-substituted 5YSZ ceramics are ionic conductors under reducing conditions with negligible contribution of electronic transport. Thus, the reduction-induced changes in conductivity are in agreement with the expected changes in the concentration of defects as described by Equations (4) and (5): a decrease in electron-hole concentration and *p*-type electronic transport and an increase in oxygen-vacancy concentration and ionic conductivity. Apparently, manganese cations remain in a 2+ oxidation state in the studied p(O_2_) range under reducing conditions resulting in p(O_2_)-independent total (ionic) conductivity. This is in agreement with the data on manganese oxidation state in doped 9.5YSZ single crystals (Mn content ≤ 0.1 wt.%) determined by combined EPR and optical absorption measurements [49,50,52,53], and also with the results of thermodynamic modeling of the Zr-Y-Mn-O system [51].

Figure 12B shows the composition dependence of ionic conductivity in Mn-substituted 5YSZ at 900 °C under reducing conditions. While oxygen vacancy concentration increases linearly with Mn content, σ_O_ goes through a maximum (2 mS × cm^−1^) at *x* ~0.10, although still being lower compared to 8YSZ (10 mS × cm^−1^ [58]). This differs from the results obtained under oxidizing conditions where ionic conductivity decreases with increasing Mn content, presumably due to the increasing impact of the defect association. All manganese is in a 2+ oxidation state under reducing conditions, and Mn^2+^ cations have a stronger tendency to form dopant-vacancy pair clusters (a lower dopant-vacancy binding energy) compared to Mn^3+^ [63]. The observed maximum of ionic conductivity for *x* = 0.10 under reducing conditions may imply the relevance of steric effects, namely lattice expansion on reduction with an impact on the oxygen-ion mobility.

**Figure 13:**
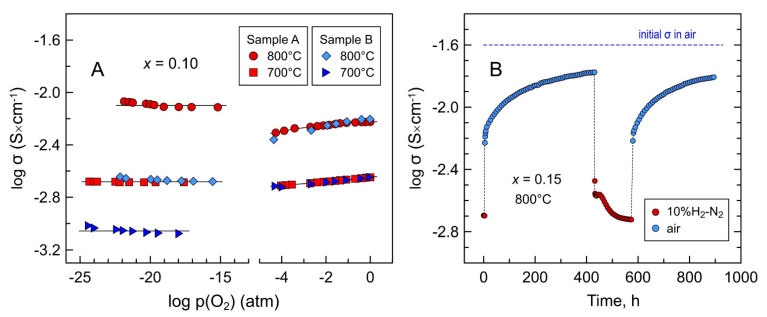
(**A**) Oxygen partial pressure dependence of electrical conductivity of *x* = 0.10 samples measured in different regimes. Sample A: measurements were done first in oxidizing and then in reducing atmospheres with reduction at 900 °C. Sample B: measurements were done first at 800 °C and then at 700 °C with reduction at given T. (**B**) Variations of electrical conductivity of the *x* = 0.15 ceramics on cycling between reducing and oxidizing atmospheres at 800 °C. The experiment was conducted after σ-p(O_2_) measurements under oxidizing and then under reducing conditions at 700–900 °C.

The electrical studies also showed that the reducibility or reduction kinetics of at least bulk samples decreases with reducing temperature down to 700–800 °C. As an example, Figure 13A compares the σ-p(O_2_) data at 700–800 °C for the *x* = 0.10 samples reduced under different conditions. The sample preliminary reduced at 900 °C was found to exhibit higher conductivity than the sample reduced at 700 or 800 °C.

**Figure 14:**
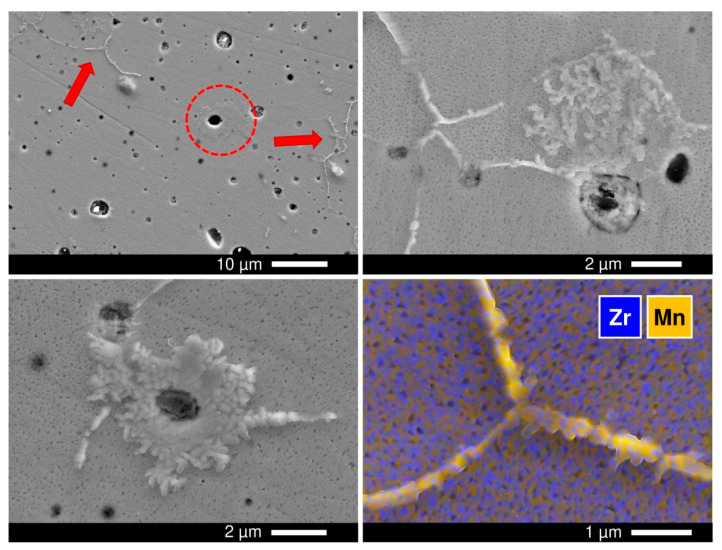
SEM images of polished *x* = 0.10 sample annealed in 10% H_2_-N_2_ flow at 900 °C for 240 h. The image in the right bottom corner is an SEM micrograph with overlaid EDS elemental mapping.

Another observation is that treatments under reducing conditions promote certain microstructural changes of the ceramics surface. SEM/EDS inspection of polished *x* = 0.10 and *x* = 0.15 samples after annealing in 10% H_2_-N_2_ flow at 900 °C for 240 h revealed the irregular accumulation of MnO*_y_* both at the grain boundaries and at the surface of grains, particularly around and inside the pores (Figure 14). Segregation of MnO*_y_* was also detected on the surface of ceramics samples after prolonged electrical measurements under reducing conditions at 700–900 °C. It should be noted that the manganese oxide exsolution under reducing conditions was rather surprising given that it was not observed under oxidizing conditions even after longer annealing (Figure 8B) and that the thermodynamic calculations predict an increase in Mn solubility on reduction [51]. Nonetheless, the experimental results imply that reducing conditions promote the exsolution of excess manganese from the fluorite lattice, even though the process is sluggish and is likely to be limited to the exposed grain boundaries and surface.

Reduction-induced microstructural changes at the surface seem to be responsible for not entirely reversible changes in electrical conductivity on cycling between oxidizing and reducing conditions. An example is shown in Figure 13B for the *x* = 0.15 ceramics at 800 °C. The sample showed an apparently reproducible variation of conductivity, with a faster decrease on reduction and slower recovery during oxidation. However, the level of conductivity dropped compared to the initial value obtained during initial measurements under oxidizing conditions. Post-mortem XRD analysis of that sample (crushed into powder) still showed the cubic fluorite structure with no evidence of phase impurities.

### 3.5. Thermochemical Expansion

**Figure 15:**
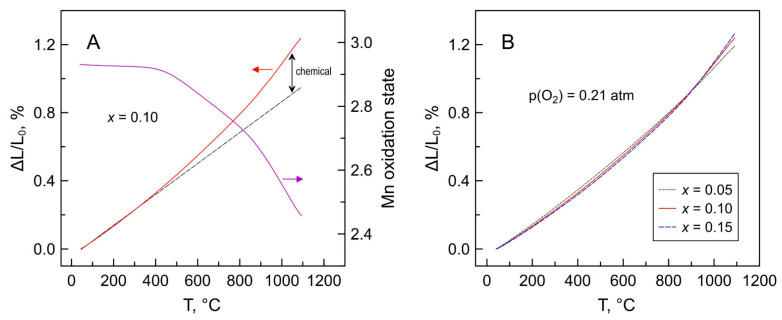
(**A**) Relative elongation of the *x* = 0.10 ceramics in air and corresponding changes in average Mn oxidation state; and (**B**) dilatometric curves of Mn-substituted 5YSZ ceramics in air.

The dilatometric curves of Mn-substituted 5YSZ ceramics in air exhibit a non-linear behavior, and can be approximated by three segments in the studied temperature range (Figure 15A). In the low-temperature range below ~400 °C, when the oxygen exchange with the gas phase is frozen, the observed expansion of oxide materials corresponds to the “true” thermal expansion of the lattice originating from the anharmonicity of atomic vibrations. Increasing temperature gives rise to a “chemical” contribution to the thermochemical expansion [64,65] associated with the increase in [Mn^2+^]/[Mn^3+^] ratio on heating due to oxygen losses from the lattice and the reduction of Mn cations, and consequently an increase in their average ionic radius. The changes in the slope of dilatometric curves reflect the corresponding inflections in temperature dependencies of the oxygen nonstoichiometry and the mean manganese valence (Figure 15A).

In the low-temperature range, the average linear thermal expansion coefficients (TECs) of the Mn-substituted 5YSZ ceramics decrease slightly with increasing manganese content (Figure 15B and Table 6), in correlation with changes in the lattice parameter (Figure 3). On the contrary, average TEC values increase with Mn content in the high-temperature range, particularly above ~850 °C, due to the increasing contribution of the chemical expansion. Still, the average thermal expansion coefficients of prepared Mn-substituted 5YSZ ceramics at 25–1100 °C (Table 6) is comparable to that of the 8YSZ solid electrolyte (10.7–10.9 ppm/K [66,67]), while somewhat excessive expansion at higher temperatures may be useful for buffer interlayers to bridge the gap between TECs of zirconia-based solid electrolyte and oxygen electrode materials including classical (Ln,A)MnO_3+δ_ (10–13 ppm/K [67,68,69]), state-of-the-art (La,Sr)Co_0.2_Fe_0.8_O_3−δ_ (LSCF) (average 14.8–15.4 ppm/K [69] but up to 22.0–24.5 ppm/K at 700–1100 °C [70]) or layered Ruddlesden-Popper Ln_2_NiO_4−δ_-based nickelates (12–15 ppm/K [71]).

**Figure 16:**
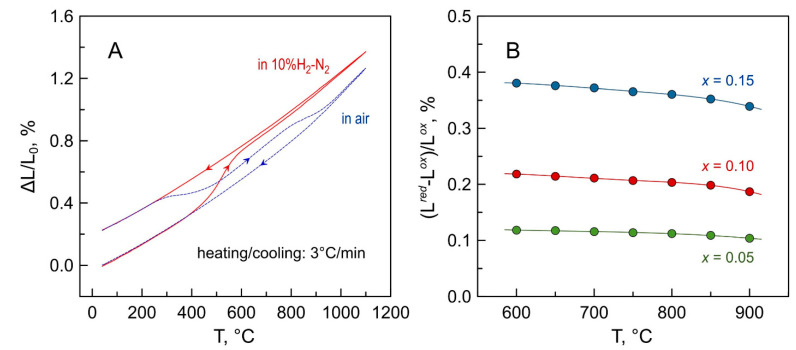
(**A**) Dimensional changes of oxidized *x* = 0.10 ceramics in one heating/cooling cycle in 10% H_2_-N_2_ atmosphere followed by a heating/cooling cycle in air; and (**B**) Temperature dependence of chemical expansion of oxidized Mn-substituted 5YSZ ceramics on reduction calculated from the dilatometric data on cooling in corresponding atmospheres.

Heating the Mn-substituted 5YSZ ceramics in a reducing atmosphere results in a noticeable expansion between 400 and 600 °C caused by Mn^2+/3+^→Mn^2+^ reduction (Figure 16A). After reduction, the materials show moderate dimensional changes on thermal cycling in a 10% H_2_-N_2_ atmosphere with average TECs at 25–1100 °C even somewhat lower compared to that in air (Table 6), due to the constant 2+ oxidation state of manganese cations under reducing conditions. The dimensional changes were reversible or nearly reversible on re-oxidation. Interestingly, the contraction of the reduced ceramics on heating in air occurs in two steps, in correlation with the oxygen nonstoichiometry variations in air (Figure 5), once again implying different oxygen exchange/diffusion kinetics and/or different defect structure in Mn-substituted zirconia at temperatures ≤ 800 °C and ≥900 °C.

Figure 16B shows the linear chemical expansion of Mn-substituted 5YSZ ceramics on reduction estimated from the dynamic dilatometric data on cooling in the corresponding atmospheres, and defined as (L*^red^*− L*^ox^*)/L*^ox^* where L*^ox^* and L*^red^* are linear dimensions of the sample in oxidized and reduced states at a given temperature, respectively. Chemical expansion naturally increases with manganese additions, thus increasing the risk of stresses at the electrolyte/buffer layer interface in a hypothetical SOC configuration when Mn-substituted 5YSZ is applied as an interlayer onto the 8YSZ electrolyte at the fuel side. At the same time, the dimensional changes on the reduction of *x* = 0.10 ceramics are comparable to those of fuel electrode materials such as La(Sr)Cr(Mg,Fe)O_3−δ_ [72] or highly reduced Sr_0.85_LnTiO_3−δ_ [73] under similar conditions. Furthermore, the chemical expansion of even *x* = 0.15 ceramics is ~3 times lower compared to gadolinia-doped ceria Ce_1−*x*_Gd*_x_*O_2−δ_ (*x* = 0.1–0.2), a conventional buffer layer material which exhibits a linear expansion of up to ~1.2% on reducing p(O_2_) from atmospheric to ~10^−20^ atm at 900 °C [74,75].

## 4. Conclusions

(ZrO_2_)_0.95_(Y_2_O_3_)_0.05_]_1−*x*_[MnO*_y_*]*_x_* (*x* = 0.05, 0.10 and 0.15) ceramics with cubic fluorite structure were prepared by solid-state synthesis and sintered in air at 1600 °C. TGA complemented by XPS studies showed that Mn cations are in a mixed 2+/3+ oxidation state under oxidizing conditions. The prepared materials exhibit variable oxygen nonstoichiometry on thermal cycling in air and on isothermal redox cycling between oxidizing and reducing atmospheres, and the extent of oxygen content variations increases with the Mn concentration. As-prepared Mn-substituted 5YSZ ceramics were found to undergo a slow oxidation under oxidizing conditions at 900 °C, reflected by oxygen uptake, lattice shrinkage and changes in electrical properties. All the prepared materials show a lower total electrical conductivity compared to conventional 8YSZ solid electrolyte. Substitution by Mn gradually suppresses ionic conductivity, presumably due to defect association in the lattice, but increases *p*-type electronic conductivity under oxidizing conditions. This leads to a gradual transformation from a predominantly ionic conductor for *x* = 0.05 to a mixed conductor with equal contributions of ionic and electronic transport for *x* = 0.10 to material with prevailing electronic conduction for *x* = 0.15. The reduction in 10% H_2_-N_2_ atmosphere at 900 °C results in Mn^2+/3+^→Mn^2+^ transformation accompanied by the lattice expansion, an increase in ionic conductivity and suppression of electronic transport. All studied Mn-substituted 5YSZ ceramics are ionic conductors under reducing conditions. Thermogravimetric and dilatometric studies demonstrated a reversible behavior in relatively short cycles between reducing and oxidizing atmospheres. Longer treatments under reducing conditions cause microstructural alterations at the surface of the bulk samples, with evidence of MnO exsolution, resulting in a not entirely reversible variation of the electrical conductivity on redox cycling. The Mn-substituted 5YSZ ceramics demonstrate an average thermal expansion coefficient similar to that of 8YSZ, although Mn additions gradually increase unfavorable chemical contribution to thermochemical expansion in air and dimensional changes on reduction.

Considering different factors such as the extent of oxygen nonstoichiometry variations (oxygen storage capacity), levels of ionic and electronic conductivity and thermochemical expansion, Mn-substituted 5YSZ with 0.05 ≤ *x* < 0.10 seems to be the most suited for the application as a thin interlayer between electrolyte and oxygen electrode of reversible SOCs.

## Figures and Tables

**Table 1:** Lattice parameters and density of as-prepared [(ZrO_2_)_0.95_(Y_2_O_3_)_0.05_]_1−*x*_[MnO*_y_*]*_x_* ceramics.

*x*	Formula Unit	*a*, Å ^1^	Density, g/cm^3^	Relative Density, % ^2^
0.05	Zr_0.862_Y_0.091_Mn_0.048_O_2−δ_	5.11840(7)	5.35	90
0.10	Zr_0.818_Y_0.086_Mn_0.096_O_2−δ_	5.10632(5)	5.36	91
0.15	Zr_0.775_Y_0.082_Mn_0.144_O_2−δ_	5.09522(7)	5.40	94

^1^ The uncertainties given in parentheses are the standard deviations in the refined parameters calculated by FullProf; ^2^ Theoretical density was calculated taking into account oxygen content determined by thermogravimetric analysis (TGA).

**Table 2:** Binding energies (eV) for Mn 2*p* and Mn 3*s* core level components of XPS spectra of as-prepared Mn-substituted 5YSZ.

*x*	Mn 2*p*				Mn 3*s*		
	2*p*_1/2_	2*p*_3/2_	FWHM (2*p*_3/2_) ^1^	Δ(2*p*_3/2_−2*p*_1/2_)	3*s*(1)	3*s*(2)	Δ(3*s*(2)−3*s*(1))
0.05	653.0	641.0	3.31	12.0	82.7	89.3	6.4
0.10	653.1	641.4	3.10	11.7	83.4	89.2	5.9
0.15	652.9	641.2	3.03	11.7	83.1	89.2	6.1

^1^ Full width at half maximum (FWHM) was obtained assuming a single contribution to the Mn 2*p*_3/2_ component.

**Table 3:** Parameters of Arrhenius model ^1^ for the total electrical conductivity of as-prepared [(ZrO_2_)_0.95_(Y_2_O_3_)_0.05_]_1−*x*_[MnO*_y_*]*_x_* ceramics in air (340–970 °C).

*x*	E_A_, kJ/mol	ln(A_0_)	ρ ^2^
0.05	96.1 ± 0.2	13.02 ± 0.03	0.99996
0.10	80.8 ± 0.7	11.14 ± 0.10	0.9991
0.15	72.2 ± 0.3	10.76 ± 0.04	0.9998

^1^ σ = (A_0_/T)exp(−E_A_/(RT)); ^2^ ρ is the correlation coefficient.

**Table 4:** Observed E_exp_/E_th_ values and oxygen-ion transference numbers obtained by the modified electromotive force (EMF) technique before and after relaxation (>70 h) at 900°C under air/O_2_ gradient.

*x*	Before Relaxation		After Relaxation		R_after_/R_ini_^1^
	E_exp/_E_th_	$\bar{t_{O}}$	E_exp/_E_th_	$\bar{t_{O}}$	
0.05	0.984	0.990	0.957	0.968	1.83
0.10	0.52	0.62	0.42	0.50	0.86
0.15	0.47	-	0.13	0.14	0.59

^1^ R_after_ and R_ini_ are the values of ohmic resistance of the sample after and before relaxation, respectively.

**Table 5:** Oxygen-ion transference number obtained by the modified EMF technique using air/(10% H_2_-N_2_) concentration cells.

T, °C	*x* = 0.05		*x* = 0.10		*x* = 0.15	
	log *p*_1_ (atm) ^1^	$\bar{t_{O}}$	log *p*_1_ (atm)	$\bar{t_{O}}$	log *p*_1_ (atm)	$\bar{t_{O}}$
900	−19.4	0.9991	−18.2	0.983	−17.4	0.997
850	−20.4	0.9995	−19.3	0.991	−18.4	0.997
800	−21.2	0.9996	−20.5	0.992	−19.3	0.997
750	−21.4	0.9997	−21.6	0.990	−20.2	0.996
700	−21.5	0.9996	−23.0	0.986	−20.9	0.995

^1^*p*_1_ is the oxygen partial pressure in 10% H_2_-N_2_ flow measured by oxygen sensor.

**Table 6:** Average thermal expansion coefficients ^1^ of Mn-substituted 5YSZ ceramics under oxidizing and reducing atmospheres.

Atmosphere	T Range, °C	Average Linear Thermal Expansion Coefficient $(\bar{\alpha }$ ± 0.1) × 10^6^, K^−1^		
		*x* = 0.05	*x* = 0.10	*x* = 0.15
air	40–1100	10.9	11.1	11.2
	40–400	9.5	9.0	8.7
	400–850	11.4	11.7	11.8
	850–1100	13.8	16.1	17.4
10% H_2_-N_2_	40–1100	10.7	10.5	10.6

^1^ TEC values are calculated from the dilatometric data obtained on cooling in the corresponding atmosphere.

## Data Availability

Data is contained within the article and/or available from the corresponding author upon reasonable request.
